# High-pressure processing enhances saltiness perception and sensory acceptability of raw but not of cooked cured pork loins—leveraging salty and umami taste

**DOI:** 10.3389/fnut.2024.1352550

**Published:** 2024-02-15

**Authors:** Tomas Bolumar, Regina Lohmayer, Manuela Peukert, Kai Thiemann, Siegfried Münch, Dagmar A. Brüggemann

**Affiliations:** Department of Safety and Quality of Meat, Max Rubner Institute (MRI), Kulmbach, Germany

**Keywords:** high pressure processing, HPP, sensory perception, salt reduction, partition coefficient, umami, meat products

## Abstract

The salt (NaCl) content in processed meats must be reduced because of its adverse effects on cardiovascular health. However, reducing salt in meat products typically leads to a lower taste intensity and, thus, consumer acceptability. Industry interventions must reduce salt content while maintaining taste, quality, and consumer acceptability. In this context, high-pressure processing (HPP) has been proposed to enhance saltiness perception, though there are contradictory reports to date. The present work aimed to conduct a targeted experiment to ascertain the influence of HPP (300/600 MPa) and cooking (71°C) on saltiness perception and sensory acceptability of meat products. HPP treatment (300/600 MPa) did enhance those two sensory attributes (approx. +1 on a 9-point hedonic scale) in raw (uncooked) cured pork loins but did not in their cooked counterparts. Further, the partition coefficient of sodium (*P*_Na+_), as an estimate of Na^+^ binding strength to the meat matrix, and the content of umami-taste nucleotides were investigated as potential causes. No effect of cooking (71°C) and HPP (300/600 MPa) could be observed on the *P*_Na+_ at equilibrium. However, HPP treatment at 300 MPa increased the inosine-5′-monophosphate (IMP) content in raw cured pork loins. Finally, hypothetical HPP effects on taste-mediating molecular mechanisms are outlined and discussed in light of boosting the sensory perception of raw meat products as a strategy to achieve effective salt reductions while keeping consumer acceptability.

## 1 Introduction

High sodium (Na) intake is associated with high blood pressure, and reducing salt (NaCl) consumption to 5 g/day could mitigate cardiovascular diseases by 24% (1). Nearly 20% of Na-intake comes from processed meats (2). Hence, reformulating strategies such as the use of salt replacers (e.g., KCl), flavor enhancers and water binders’ ingredients, and processing methods have been extensively researched in processed meats (3–7). However, the multi-functionality of salt in the texture, flavor, and microbial shelf-life presents a challenge. Thus, there are still practical limitations to overcome, and one of the most remarkable is a weakening of the perception of saltiness and overall flavor intensity in salt-reduced products. This usually leads to reduced taste intensity and consumer acceptability, making its industrial implementation difficult (8).

Application of HPP serves as a cold pasteurization in meat products (9, 10) and has been proposed to compensate for the effects of salt reduction in ready-to-eat meat products (11). Moreover, the application of HPP to meat products has also been associated with an increased saltiness perception (12–15). However, contradictory results have been reported in the literature, with HPP treatment not affecting saltiness perception (6, 16–18). The weakening of the interaction between ions and muscle proteins may be a reason for that. HPP treatment will denature proteins, modifying electrostatic interactions between ions and muscle proteins. Consequently, releasing additional ions from the meat matrix (for instance, Na^+^) could be favored, increasing this way saltiness perception. A relationship exists between sodium concentration in the saliva and perceived saltiness (19). The sodium concentration in the mouth should depend on sodium’s binding strength to the meat matrix and its solubilization into the water phase. Determining the partition coefficient (P) can estimate this physicochemical interaction. The higher the *P*_Na+_, the higher the proportion of salt in the water phase (saliva), potentially raising the perceived saltiness. The disassociation of NaCl in meat systems and the equilibrium established with ions bound to proteins and free ions are vital for the structure of the meat product and the perception of saltiness. The denaturation of proteins will change the native tridimensional structure of the proteins, whereby changing the molecular interactions of the polar and non-polar groups at the surface and interphases of the protein/−s with the water and the ions in the surrounding molecular system (20). Due to the thermal treatment applied to cooked meat products, muscle proteins are largely in a denatured state, and consequently, modification of protein structure in that case, for instance, using HPP, to affect the content of ions bound to proteins must be already minimal. In contrast, we hypothesized that meat products not undergoing a thermal treatment must be more susceptible to modification of their ionic equilibrium with the muscle proteins. Therefore, both raw and cooked cured pork loins were included in this study.

Besides, umami substances could act as flavor enhancers, especially relevant in salt-reduced foods. Nucleotides present in meat such as adenosine-5′-monophosphate (AMP), inosine-5′-monophosphate (IMP), and guanosine-5′-monophosphate (GMP) are umami-taste compounds (21), which also function as flavor enhancers (22, 23). They work synergistically with amino acids and intensify the taste sensation by binding to the same taste receptors (24, 25). HPP has recently been proven to modify the concentration of different nucleotides in meat by affecting endogenous enzyme activities under pressure conditions, potentially resulting in the accumulation of umami-taste compounds (26, 27).

The aim of this study was: firstly, to assess the effect of an HPP treatment at 300 and 600 MPa on the saltiness perception and overall acceptability of raw and cooked brine-immersed cured pork loins, and secondly, to investigate the molecular basis affecting the release of sodium and the presence of umami substances derived from the nucleotide breakdown in cured pork loins as affected by processing conditions such as cooking (71°C) and HPP treatment (300/600 MPa).

## 2 Materials and methods

### 2.1 Sensory study

#### 2.1.1 Curing, cooking, and HPP treatment of pork loins

Pork loins, *m. longissimus* (*n* = 6) were collected from a local processing plant (Kulmbach slaughterhouse) and processed within 24–48 h post-mortem. Selected loins were within a pH range of 5.40–5.55. Loins were salted by immersion into an appropriate brine prepared using curing salt [99.5 (g/100 g) NaCl and 0.5 (g/100 g) NaNO_2_] to reach an approximate concentration of 2 (g/100 g) NaCl in the final product. Similar meat products can be found commercially available, and microbiological safety is guaranteed and validated when working with good manufacturing practices. Loins were immersed in the brine for three weeks at 2°C. After salting, loins were split longitudinally into halves. Three left half-sides and three right half-sides were allocated to each raw or cooked group. The cooking was done by submersing the vacuum-packaged halves (PA/PE 90 bags) in a water bath at 74°C to an internal temperature of 71°C. The day after, all the loin halves were cut into slices of 2 mm, giving approximately 200 slices. Then, individual successive slices along the longitudinal direction of the loin half were distributed into three subgroups (i.e., each loin around 65 slices per treatment) for further processing, namely: no application of HPP treatment (untreated control at atmospheric pressure = 0.1 MPa), and application of HPP treatment at 300 MPa or 600 MPa for 5 min at room temperature (EPSI high-pressure system, Belgium). Packages with slices of cured pork loins allocated to the same sensory session were HPP-treated together in order to minimize the potential effect of the particular HPP conditions on product quality.

#### 2.1.2 Sensory analysis

Six sensory sessions were conducted with a product of 3–5 storage days, along 3 consecutive days. Each sensory session included six different product samples, corresponding to two different loins (either raw or cooked), with each loin treated at three different pressure levels (0.1, 300, and 600 MPa). Three slices per loin and treatment were given to each panelist for sensory evaluation. Sensory evaluations of raw and cooked cured pork loins were done separately. Panelists wore red UV glasses (Solarium safety red UV glasses with elastic, 600,015-red, Wellnessprofi) in order to fade color differences between the various cured loins treated at different high-pressure levels, avoiding this way misleading sensory scores stemming from color differences of the product. A reference product, a counterpart with the same characteristics for both raw and cooked meat products, previously purchased in a local supermarket, was presented to the panelists before starting the sensory test, and a score for saltiness perception and overall acceptability for the reference sample was consensually agreed-upon testing of the reference sample among the participant panelists. Sensory evaluation was done using a scale ranging from 1 (no salty) to 9 (very salty) for saltiness perception and from 1 (extremely dislike) to 9 (extremely like) for overall acceptability. The overall acceptability was the pondering of the impressions of the product as a whole during its consumption (summing up the perceived sensations, mainly regarding taste (correct flavor and absence of off flavors), texture (bite and disruption of product in mouth), and juiciness of the product). Panelists were a group of people (*n* = 10) trained to assess meat products usually (10–20 times per year). The same group of panelists was used throughout the entire sensory evaluation.

### 2.2 Physicochemical molecular mechanisms study

In order to investigate potential physicochemical molecular mechanisms related to the increased saltiness perception and overall acceptability observed in raw cured pork loins after HPP, the partition coefficient of sodium (*P*_Na+_) and the content of nucleotide compounds were studied along with a basic characterization of end-product quality parameters such as water binding and color.

#### 2.2.1 Curing, cooking, and HPP treatment of pork loins

Pork loins (*n* = 6) were processed in the same way as described in the sensory study. Loins were collected from the same processing plant, and pH values were within a range of 5.40–5.55. After salting for three weeks at 2°C, loins were cut into six equivalent blocks, vacuum packaged, and randomly assigned to each treatment (six samples per treatment coming from six different loins of six independent animals). For each loin, three blocks were kept raw (R), and the other three were cooked (C) in a water bath at 74°C to an internal temperature of 71°C. After resting overnight, HPP treatment at 300 or 600 MPa for 5 min was applied to R and C loin blocks.

#### 2.2.2 Process yield

Process yields were quantified throughout the process, from raw materials to salting, cooking, and HPP treatment, by weighting loins before and after each processing step.

#### 2.2.3 Water content

Thirty grams of sea sand was weighed in evaporating glass dishes using an analytical scale (Sartorius CPA3202S, Göttingen; Germany) and pre-dried at 103°C overnight in an oven (WTC Binder ED240, Tuttlingen, Germany). Five grams of sample were weighed at room temperature (RT) in the evaporating dishes and mixed thoroughly with the sea sand. The dishes were dried for 10 min at 600 Watts in a microwave (Siemens HF23051, Germany) till no further weight loss could be observed. Before weighing the dried sample, the dishes were left to equilibrate in a desiccator at RT. The determination was carried out in duplicate. Water content was calculated by weight difference.

#### 2.2.4 Water activity (a_w_)

Samples were crushed for 5 s at 5000 rpm using a mixer GrindoMix (Retsch Gmbh, Haan, Germany). Subsequently, the minced sample was placed into a cylindrical plastic capsule (4 cm diameter × 1 cm height), avoiding air bubbles. The sample-filled capsule was inserted into an a_w_-meter (SE Aw Lab, SE Schulz Electronic, Hoehenkirchen, Germany). The sample was let equilibrate inside the a_w_-meter for 30 min at 25°C before reading the a_w_-value.

#### 2.2.5 Instrumental color

CIE L*-, a*- and b*-values and reflectance spectra were collected on day ten after HPP for objective measurement of color by using a portable spectrophotometer (CM-600d, Konica Minolta Sensing Europe, Munich, Germany) with a D65 illuminant and a 10° standard observer angle. After opening the vacuum packaging, samples were scanned at different locations in a freshly cut central area. Measurements were repeated ten times per sample, resulting in sixty scans per treatment. Calibration was carried out using a white standard plate (L* = 100) and a light trap (L* = 0). Hue angle and chroma (C*) were calculated using the following Equations 1, 2. Reflectance was recorded simultaneously from 400 nm to 700 nm at 10 nm increments.


(1) $$\mathrm{hue}=\tan^{-1}\left(b^{*}/a^{*}\right)$$

(2) $$C^{*}=\;{\left(a^{*2}+b^{*2}\right)}^{1/2}$$

#### 2.2.6 Determination of partition coefficient (*P*_Na+_)

Methodologies were adapted from elsewhere (28–30). Cylindrical blocks (2.0 cm length × 1.6 cm of diameter, ~ 3.5 g) were cut from slices of 2 cm thickness of the different pork loins using a metallic punch. Individual cylinder samples were then placed in 50 mL plastic tubes and immersed in variable volumes of bi-distilled water. Firstly, a Na^+^ selective electrode (Metrohm, Fildestadt, Germany) was used to estimate the equilibrium time (signal leveled off). Equilibrium was reached after approx. 60 h of incubation at 2°C (data not shown). Secondly, a test was carried out, including an incubation lasting for 12 days at 2°C. Equivalent meat cylinder blocks were submersed in 10 and 20 mL bi-distilled water, and individual tubes were incubated for 0, 1, 2, 5, 6, 7, and 12 days. Sodium content was determined in the product and the water phase at the specified time points using inductively coupled plasma – mass spectrometry (ICP-MS) (2.2.7). Incubation for 7 days at 2°C ensured the equilibrium was fully reached (data not shown). Thirdly, *P_Na+_* was calculated by determining the concentration of sodium in the 3-day product at time zero (start of incubation) and in the water phase at equilibrium at four mass ratios (r) of the weight of the water phase (W_Wa_) and weight of the product (W_P_) (r = W_Wa_/W_P_) after incubation at 2°C for 7 days. The weights of cylindrical block samples were precisely weighed before and after incubation. The weight of water varied at 7, 10, 14, and 18 mL, corresponding to an r-value of 2.0, 2.9, 4.0, and 5.1, respectively. The weight of water at equilibrium was calculated according to a mass balance (Eq. 3).


(3) $$\left(W_{0}^{P}+W_{0}^{Wa}\right)=\left(W_{\mathrm{eq}}^{P}+W_{\mathrm{eq}}^{Wa}\right)$$

The following mass balance (Eq. 4) was resolved for calculation:


(4) $$\begin{array}{l} \left({\left[{Na}^{+}\right]}_{0}^{P}\;x\;W_{0}^{P}\right)+\left({\left[{Na}^{+}\right]}_{0}^{Wa}\;x\;W_{0}^{Wa}\right)=\\ \left({\left[{Na}^{+}\right]}_{\mathrm{eq}}^{P}\;x\;W_{\mathrm{eq}}^{P}\right)+\left({\left[{Na}^{+}\right]}_{\mathrm{eq}}^{Wa}\;x\;W_{\mathrm{eq}}^{Wa}\right)\; \end{array}$$

where; W = weight, P = product, Wa = water phase, 0 = time zero, eq = time at equilibrium.

Assumptions:

${\left[{Na}^{+}\right]}_{0}^{Wa}=0$ the ultrapure water used in the experiment was analyzed by ICP-MS, and the sodium content was below the detection limit of 3.84 mg/L. For calculation purposes, it was assumed to be zero.

${\left[{Na}^{+}\right]}_{\mathrm{eq}}^{P}$ was calculated by resolving the Eq. 4.

*P*_Na+_ is defined as Eq. 5:


(5) $$P=\frac{{\left[{Na}^{+}\right]}_{\mathrm{eq}}^{Wa}}{{\left[{Na}^{+}\right]}_{\mathrm{eq}}^{P}}$$

#### 2.2.7 Sodium quantification

Sodium content was determined using ICP-MS. For analysis, 0.5 g of chopped meat sample was digested in duplicate after the addition of 4 mL ultrapure water, 6 mL 65% nitric acid (14.3 mol/L), and 1 mL 30% hydrochloric acid (9.4 mol/L) in a microwave (Start 1,500, MLS GmbH, Leutkirch, Germany) at 200°C for 30 min. Digests were made up to a final volume of 25 mL with ultrapure water and further diluted 1:10 with ultrapure water prior to analysis. For analysis of the water phase, liquid samples were filtered (0.45 μm, cellulose acetate, Sartorius Stedim Biotech GmbH, Göttingen, Germany) and diluted in duplicate 1:200 with 2% nitric acid solution (0.3 mol/L). Analysis of sodium was done using ICP-MS equipment (Agilent 7,800, Agilent Technologies, Waldbronn, Germany). Argon was used as a sample injection and plasma and aerosol dilution gas. Helium was used as collision gas. The acid matrix of the calibration standards was matched to the matrix of the digested meat samples and the diluted liquid samples, respectively. Internal standards were added online to the sample flow.

#### 2.2.8 Nucleotide quantification

Umami-stimulating nucleotides IMP and GMP and the IMP precursor, AMP, were determined by liquid chromatography coupled to a mass spectrometer (LC–MS). For sample preparation, one g of frozen meat sample was extracted with 10 mL ice-cold 80:20 methanol:water (v/v) using a bead mill homogenizer (Bead Ruptor Elite, Omnilab, Germany). After homogenization, samples were incubated at −20°C for 30 min. The raw extract was centrifuged, and the pellet was re-extracted with 10 mL ice-cold 80:20 methanol:water (v/v) by repeating the first extraction step. Both supernatants were combined and mixed. One ml of the combined extract was again centrifuged (15,000 × g for 20 min, 4°C), and the supernatant was stored at −80°C until analysis. A DionexUltiMate 3,000 RS HPLC from Thermo Scientific (Waltham, USA) coupled to a maXis UHR-QToF system (Bruker Daltonik, Bremen, Germany) was used for the analysis. The column was a HILIC TSKgel Amide-80, 5 μm, 4.6 mm I.D. × 10 cm (Tosoh Bioscience GmbH, Griesheim, Germany). The column temperature was set at 40°C, and the injection volume was 15 μL. The mobile phase consisted of eluent A (95:5 water: acetonitrile with 20 mM ammonium acetate) and eluent B (90:10 acetonitrile:water with 20 mM ammonium acetate). The flow rate was 300 μL/min, and the step gradient was as follows: an initial 2 min at 10% A; 2–3 min to 60% A; elution of compounds during a 3–9 min isocratic step at 60% A; 9–10 min to 30% A for flushing and hold at 30% A from 10–13 min. After flushing, the gradient returned to starting conditions (10% A) from 13–16 min, and the column was re-equilibrated at 10% A for 5 min. Instrument settings for MS analysis were: positive ion mode [M–H]^+^, capillary voltage 4.5 kV, nebulizer gas 5 bar, desolvation gas flow at 10 L/min, and a desolvation gas temperature of 250°C. MS data were acquired using Compass software 1.7 (Bruker Daltonik, Bremen, Germany). For data analysis, ion chromatograms using the exact compound masses were created, and peak areas were integrated for AMP (348.071), IMP (349.056), and GMP (364.065). Calibration was performed using triplicate runs of AMP, IMP, and GMP in the range [0.2–50 μmol/L].

### 2.3 Statistical analysis

ANOVA of three factors was conducted to assess the effect of panelist (*n* = 10), loin (*n* = 6), and pressure level (*n* = 3; 0.1, 300, and 600 MPa). Raw and cooked cured pork loins were analyzed separately for the variables, saltiness perception, and overall sensory acceptability. The statistical software package, JMP Software, from SAS was used. A one-way analysis of variance (ANOVA) with Tukey’s pairwise test was used to analyze the statistical differences between processing treatments and storage times using the software Past version 3.2.

## 3 Results and discussion

### 3.1 Sensory study

Earlier sensory tests with cooked ham treated by HPP at 600 MPa showed no differences in the saltiness perception compared to a control (untreated) (data not shown). In the present study, both raw and cooked brine-immersed cured pork loins were included to investigate different extents of protein denaturation, which, in turn, likely have different electrostatic interactions. This electrostatic-interaction differential could result in different perceived saltiness. The test was conducted with loins cured by immersion (to minimize mechanical muscle disruption) and with no addition of phosphates (to avoid any other chemical interactions).

The salting process resulted in a product weight gain of 10.2 ± 0.6 (g/100 g). The storage of the raw cured pork loins for two days before slicing resulted in a weight loss of 2.8 ± 0.2 (g/100 g) due to water stabilization inside the loins. The cooking process resulted in a cooking loss of 27.5 ± 0.6 (g/100 g). Noteworthy, raw cured pork loins had a higher content of salt (NaCl) (~2.1 (g/100 g), namely 2.16, 2.10 and 2.08 for 0.1, 300 and 600 MPa, respectively) than cooked cured pork loins [~1.9 (g/100 g), namely 1.85, 1.88 and 1.90 for 0.1, 300 and 600 MPa, respectively]. The cooking process expelled water but also salts, so the resulting cooked products had a lower concentration of salt than the raw products (*p* < 0.05). No differences in the salt content were observed with pressure treatment and pressure level.

**Figure 1:**
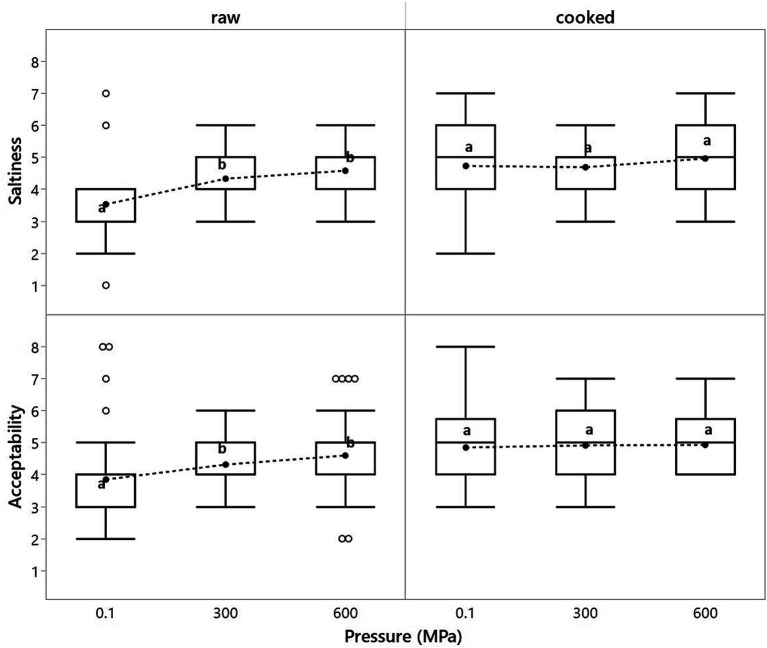
Effect of high-pressure treatment at 300 and 600 MPa for 5 min on saltiness perception and overall acceptability of raw (uncooked) and cooked brine-immersed cured pork loins (*n* = 6). Different superscripts within the same sensory trait and subjected to thermal processing or not (either raw or cooked) mean a significant difference at *p* < 0.05.

Figure 1 summarizes the sensory results regarding saltiness perception and overall acceptability of raw and cooked brine-immersed cured pork loins, and subsequently, subjected to HPP at 300 and 600 MPa. HPP treatment at 300 and 600 MPa increased the saltiness perception and overall acceptability of raw cured pork loins (*p* < 0.001). Control raw pork loins that were not treated by HPP scored 3.53, whereas HPP treatment at 300 and 600 MP raised this score to 4.33 and 4.58, respectively (Figure 1). This fact was translated into higher overall acceptability of the HPP-treated raw cured pork loins, with a score of 4.31 and 4.60, for 300 and 600 MPa, respectively, compared to the control (not HPP-treated) that scored 3.85 (Figure 1). Despite the higher absolute value scored in saltiness perception and overall acceptability of the raw cured pork loins treated at the highest pressure (600 MPa), the differences between the two pressure levels (300/600 MPa) were statistically not significant. In contrast, no differences in saltiness perception and overall acceptability were observed in the cooked cured pork loins treated by HPP at 300 or 600 MPa compared to the control (not HP-treated) (Figure 1), as we had already observed in earlier tests.

It is noteworthy that cooked loins ranked a slightly higher score for both saltiness perception and overall acceptability than their raw counterparts (Figure 1). This fact would support the theory that cooked meat products are generally perceived as saltier than their raw counterparts even though cooked had indeed a significantly lower salt content [1.87 (g/100 g)] than raw cured pork loins [2.11 (g/100 g)], which agrees with the theory that cooked meat products do taste saltier than their raw counterparts (31). One of the reasons for this preference for cooked meat products over raw may stem from the fact that meat’s taste develops with cooking (22, 32, 33).

The molecular mechanisms explaining these differences in saltiness perception are not well-established. However, the most probable cause may be the extent of muscle protein denaturation, which surely must be higher in cooked than in raw meat products. It is well established that heat and high pressures lead to the denaturation of proteins, and using differential scanning calorimetry (DSC), the T/P denaturation domains and level of affection have been quantified in meat products (34, 35). The application of HPP to raw meats would induce a level of protein denaturation, in effect changing the electrostatic interactions. In contrast, the HPP treatment would not result in further significant protein denaturation in a cooked product, as the muscle proteins would already be in a denatured state. Previous studies that showed increased saltiness perception after HPP treatment were primarily conducted with dry-cured ham (12–14), indeed, a raw cured meat product. Whereas in studies with cooked meat products, no saltiness enhancement with HPP was observed (6, 16–18). That strongly agrees with our hypothesis that manipulating saltiness perception through HPP is more plausible in raw (uncooked) meat products than in cooked ones. However, to the best of our knowledge, no study has included cooked products and their raw counterparts in the same study and compared their perceived saltiness in a sensory analysis, as it does the present. Enhancing saltiness perception in raw, further-processed meat products through processing interventions such as an HPP treatment represents a novel, feasible approach that could allow lower salt content.

The following section describes an investigation regarding the potential molecular physicochemical mechanisms involved in the enhanced saltiness perception observed in raw cured pork loins after HPP, as opposed to cooked cured pork loins. Classical quality properties, such as water retention and color parameters, and specifically, additional tests looking into properties such as the *P*_Na+_ and the content of umami-taste nucleotides, were carried out.

### 3.2 Physicochemical molecular mechanisms study

#### 3.2.1 Process yield, sodium content, and water binding

The cooking process resulted in a cooking loss of approx. 20 (g/100 g) (Table 1). This cooking loss also resulted in a loss of salt (NaCl) (Table 1). Again, cooked pork loins had a lower salt (NaCl) content [an average of 1.71 (g/100 g)] than their raw counterparts [an average of 1.85 (g/100 g)]. A similar pattern of water and salt losses was observed in the sensory study. HPP treatment of raw and cooked cured pork loins resulted in the expelling of exudates in the range of 1–2 (g/100 g). This value is in the same order of magnitude as the weight losses that occurred during storage of the control product (0.1 MPa) (Table 1). All cooked products had approximately 4–5 (g/100 g) less water content than their raw counterparts (*p* < 0.05) (Table 1). The a_w_-values were within a tight range of 0.974–0.980 (Table 1).

**Table 1:** Effect of cooking (T_internal core_ = 71°C) and high-pressure treatment at 300 and 600 MPa for 5 min on processing yields^#^, salt (NaCl) and water contents (g/100 g), water activity (a_w_) and color parameters (L^*^, a^*^, b^*^, C^*^, and hue) of raw and cooked brine-immersed cured pork loins (mean ± standard error) (*n* = 6).

	Raw			Cooked		
	Control	HPP		Control	HPP	
	0.1 MPa	300 MPa	600 MPa	0.1 MPa	300 MPa	600 MPa
Cooking loss (g/100 g)	--	--	--	−20.6^A^ ± 1.2	−19.8^A^ ± 1.7	−21.1^A^ ± 1.0
HPP loss (g/100 g)	−1.2^A^ ± 0.1	−1.2^A^ ± 0.1	−1.6^A^ ± 0.3	−2.0^A^ ± 0.2	−1.4^A^ ± 0.2	−1.9^A^ ± 0.3
NaCl (g/100 g)	1.81^B^ ± 0.14	1.92^BC^ ± 0.05	1.83^B^ ± 0.13	1.73^B^ ± 0.14	1.63^AB^ ± 0.10	1.77^B^ ± 0.04
Water content (g/100 g)	73.7^B^ ± 0.4	75.0^B^ ± 0.3	73.3^B^ ± 0.4	69.9^A^ ± 0.7	69.2^A^ ± 0.3	69.3^A^ ± 0.9
a_w_	0.978 ^BC^ ± 0.001	0.978^BC^ ± 0.001	0.980^C^ ± 0.001	0.974^AB^ ± 0.001	0.976^B^ ± 0.001	0.978^BC^ ± 0.001
Color parameters						
L*	56.1^A^ ± 1.6	64.1^B^ ± 0.9	69.5^C^ ± 0.8	74.2^C^ ± 0.8	73.1^C^ ± 0.6	74.1^CD^ ± 1.0
a*	7.1^A^ ± 0.5	7.6^A^ ± 0.3	7.1^A^ ± 0.2	7.0^A^ ± 0.3	7.6^A^ ± 0.2	7.1^A^ ± 0.5
b*	10.1^A^ ± 0.4	9.1^AB^ ± 0.4	8.9^B^ ± 0.4	8.3^B^ ± 0.1	8.7 ^B^ ± 0.2	8.5^B^ ± 0.2
C*	12.4^A^ ± 0.5	11.9^AB^ ± 0.5	11.4^AB^ ± 0.3	10.9^B^ ± 0.2	11.5^AB^ ± 0.3	11.1^AB^ ± 0.4
Hue	55.2^A^ ± 2.0	50.1^AB^ ± 1.2	51.2^AB^ ± 1.2	50.1^AB^ ± 0.8	48.8^B^ ± 0.5	50.5^AB^ ± 1.3

Different superscripts within the same row mean a significant difference at *p* < 0.05. ^#^The salting process had a process yield or brine uptake of 7.7 ± 0.39 (g/100 g) (mean ± standard error) (*n* = 6).

#### 3.2.2 Instrumental color

**Figure 2:**
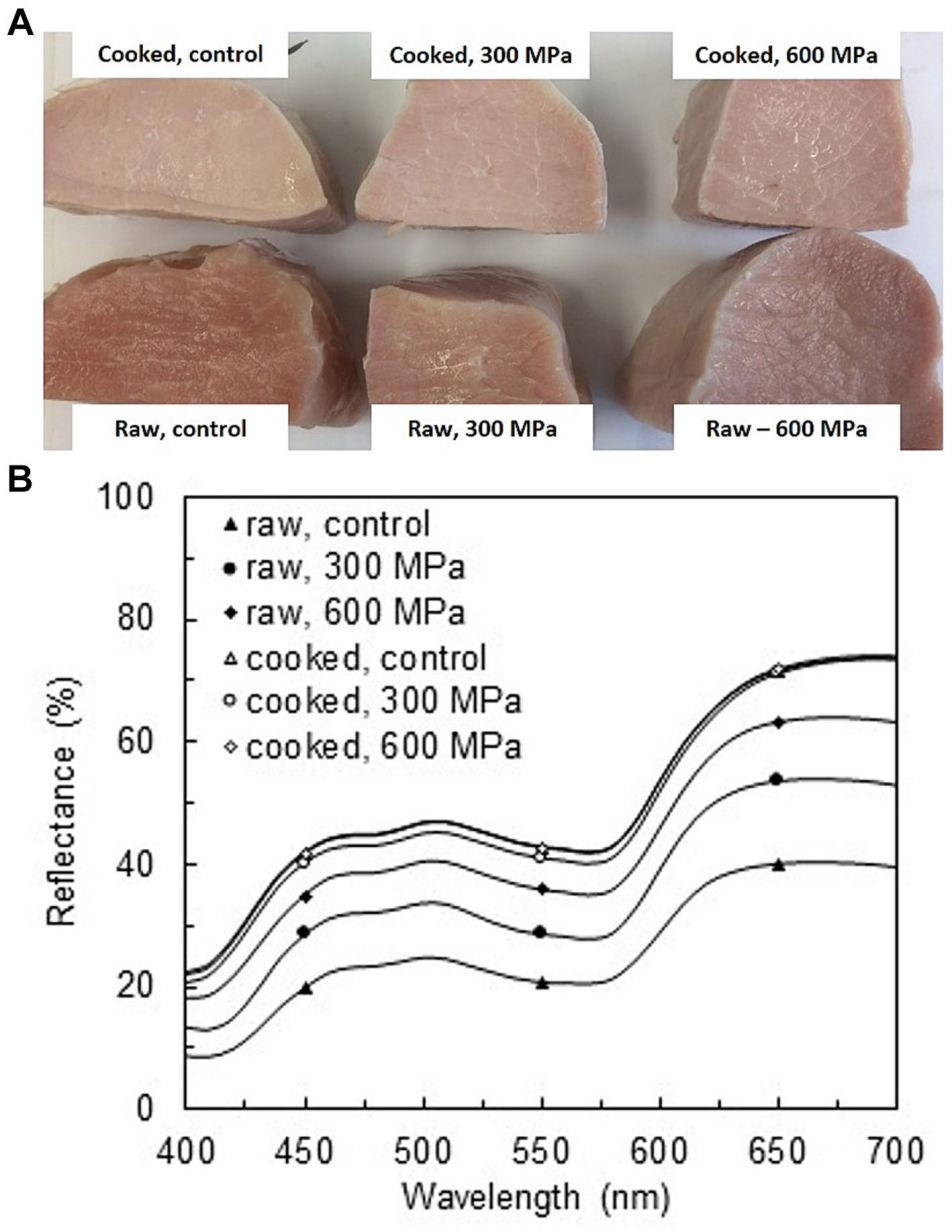
Illustrative picture **(A)** and corresponding reflectance spectra **(B)** of the raw (uncooked) and cooked brine-immersed pork cured loins, untreated (control) or subsequently treated by high-pressure processing (HPP) at 300 or 600 MPa (*n* = 6).

HPP impacted the color of meat products (Figure 2). In general, changes in L* (lightness) are observed already at pressures of 200 MPa, whereas a^*^ (redness) decreases with pressures higher than 300–400 MPa (36). Several studies have reported a threshold of around 350–400 MPa for the lightness effect. Accordingly, above 400 MPa, no further increase in lightness can be measured (37). However, when the product is cured, nitrification protects myoglobin from oxidation, and changes of redness (a^*^) are minimal, with changes in lightness (L^*^) due to protein denaturation the most notorious effect (36). These theoretical patterns of HPP-induced color changes matched in a satisfactory manner to our results (Table 1), where the most remarkable color change was for the L*-value. Interestingly, the increase in L^*^ followed the same pattern as the perceived saltiness intensity (in an increasing order; Raw-Control < Raw-HPP-treated < Cooked), indirectly pointing out a relationship between protein denaturation and perceived saltiness intensity.

Reflectance is the quantification of the radian energy reflecting on the surface. It relates to the L*-value in meat products and can also be associated with the protein denaturation extent. Figure 2 shows that increased pressure (600 MPa > 300 MPa > 0.1 MP) in raw cured pork loins increased reflectance. This observation is in line with the conclusions from Table 1 on color, particularly with the L*-value and the saltiness perception order of R-600 MPa = R-300 MPa > R-0.1 MPa, where a higher reflectance can be associated with a higher saltiness perception. Furthermore, cooked products had higher reflectance than their raw counterparts (Figure 2). However, all cooked pork loins, non-HPP-treated (0.1 MPa) or HPP-treated at 300 or 600 MPa, had similar reflectance spectra. This fact would also support the absence of a different saltiness perception among the different cooked products, i.e., treated or not with HPP, and would support the hypothesis that HPP treatment of cooked pork loins will not induce a significant further protein denaturation, and consequently, no effect on the perceived saltiness.

#### 3.2.3 Partition coefficient (*P*_Na+_)

Table 2 shows the effect of HPP at 300 and 600 MPa for 5 min on the *P*_Na+_ from raw and cooked brine-immersed cured pork loins. The higher the *P*_Na+_, the higher the proportion of salt in the water phase (saliva), potentially raising the perceived saltiness. No differences were observed for the value of the *P*_Na+_ among the different treatments assayed (i.e., raw or cooked, and application of HPP treatment at 300 or 600 MPa). No differences were observed even when the statistical comparison was made, grouping all the values into two main categories: raw (*P*_Na+_ = 0.99 **±** 0.18) and cooked cured pork loins (*P*_Na+_ = 1.06 **±** 0.17). Nonetheless, it can generally be stated that cooked cured pork loins were perceived as saltier than uncooked cured pork loins (Figure 1), despite actually having a lower salt (NaCl) content [2.11 (g/100 g) in raw versus 1.88 (g/100 g) in the cooked product]. A rule of thumb in meat science claims that cooked meat products are generally saltier than their raw counterparts (31). Although, no solid experimental data describe the responsible mechanisms. Ruusunen, Simolin, and Puolanne (38) showed that replacing lean pork meat with pork fat increased the sausage’s perceived saltiness. When water was replaced with pork fat on an equal weight basis, the sausage’s perceived saltiness did not change. These authors reported a strong negative correlation (*p* < 0.01) between perceived saltiness and protein content, thus suggesting a causative link between these two factors. The same effect was also described in another study where lowering fat and increasing meat proportion resulted in meat products that tasted less salty (39). Protein content and its ability to establish electrostatic interactions could be a major determinant of sodium binding within the meat matrix and the perceived saltiness. Because there was a significantly higher saltiness perception for the HPP-treated raw cured pork loins, a more plausible explanation would come from the kinetics of the release of Na^+^ in the short time course of events (i.e., during mastication), which could likely be more determinant for the saltiness perception than the concentration of sodium at the equilibrium (as calculated for the *P*_Na+_).

**Table 2:** Effect of high-pressure treatment at 300 and 600 MPa for 5 min on the partition coefficient of sodium ion (P_Na+_) of raw (uncooked) and cooked brine-immersed cured pork loins (mean ± standard error) (*n* = 6).

	Raw			Cooked		
	Control	HPP		Control	HPP	
r^#^	0.1 MPa	300 MPa	600 MPa	0.1 MPa	300 MPa	600 MPa
2.0	1.00^A^ ± 0.06	0.91^A^ ± 0.06	0.92^A^ ± 0.10	1.04^A^ ± 0.08	1.08^A^ ± 0.05	0.95^A^ ± 0.07
2.9	1.14^A^ ± 0.17	1.01^A^ ± 0.32	0.98^A^ ± 0.12	1.13^A^ ± 0.12	1.31^A^ ± 0.22	0.95^A^ ± 0.10
4.0	0.92^A^ ± 0.12	0.87^A^ ± 0.19	1.15^A^ ± 0.28	1.27^A^ ± 0.16	1.23^A^ ± 0.34	0.85^A^ ± 0;06
5.1	1.11^A^ ± 0.23	0.91^A^ ± 0.27	0.95^A^ ± 0.21	1.26^A^ ± 0.24	0.85^A^ ± 0.10	0.82^A^ ± 0.15
average	1.05^A^ ± 0.12	0.92^A^ ± 0.19	1.00^A^ ± 0.14	1.17^A^ ± 0.13	1.12^A^ ± 0.16	0.89^A^ ± 0.08

Different superscripts within the same row mean a significant difference at *p* < 0.05. ${\;}^{\#}r=\;\frac{\mathrm{weight}\;\mathrm{water}\;\mathrm{phase}}{\mathrm{weight}\;\mathrm{product}}$ .

#### 3.2.4 Nucleotide determination

ATP is rapidly metabolized upon slaughter into adenosine diphosphate (ADP) and AMP. Since its regeneration route is broken up, the pathway goes only downstream, forward the degradative direction, forming IMP, inosine, hypoxanthine, xanthine, and ending with uric acid (21, 22, 33).

Nucleotides quantified enhance the umami flavor in this order GMP > IMP > AMP (40). Table 3 shows the concentration of umami-taste nucleotides of raw and cooked brine-immersed cured pork loins. Cooking and HPP treatment at 600 MPa increased the content of AMP (Table 3) (*p* < 0.05). That could be due to thermal activation of the conversion from ADP to AMP, which would agree with the higher amount of ADP found in the raw cured pork loins that were not HPP-treated. Furthermore, AMP was degraded over storage time (day 3 to 22) (Table 3), suggesting that AMP-degrading enzymes are still active, at least partially, after cooking and HPP treatment.

**Table 3:** Effect of high-pressure treatment at 300 and 600 MPa for 5 min on the concentration (nmol/g of product) of umami-taste nucleotides (AMP, IMP, and GMP)^#^ of raw (uncooked) and cooked brine-immersed cured pork loins (mean ± standard error) (*n* = 6).

	Storage time (days)	Raw			Cooked		
		Control	HPP		Control	HPP	
		0.1 MPa	300 MPa	600 MPa	0.1 MPa	300 MPa	600 MPa
AMP	3	0.6^A.a^ ± 0.01	0.7^A.a^ ± 0.06	3.8^B.a^ ± 0.42	7.3^C^˙^a^ ± 0.23	6.8^C^˙^a^ ± 0.56	7.1^C^˙^a^ ± 0.58
	22	0.5^A.b^ ± 0.01	0.5^A.b^ ± 0.01	0.5^A.b^ ± 0.02	4.9^B.b^ ± 0.27	5.00^B.a^ ± 0.87	6.3^B.b^ ± 0.30
IMP	3	29.9^B.a^ ± 3.17	40.5^A.a^ ± 2.24	28.6^B.a^ ± 3.13	25.6^B.a^ ± 1.06	25.3^B.a^ ± 3.03	30.4^B.a^ ± 4.09
	22	9.2^A.b^ ± 0.81	11.9^A.b^ ± 0.82	21.5^B.a^ ± 2.14	27.1^B.a^ ± 1.33	22.5^B.a^ ± 4.41	27.1^B.a^ ± 1.63
GMP	3	1.4^A.a^ ± 0.03	1.3^AB.a^ ± 0.03	1.2^B.a^ ± 0.04	1.1^BC^˙^a^ ± 0.02	1.0^BC^˙^a^ ± 0.06	1.1^B.a^ ± 0.04
	22	0.8^A.b^ ± 0.04	0.8^AB.b^ ± 0.02	0.9^AB.b^ ± 0.03	1.1^B.a^ ± 0.02	0.9^AB.a^ ± 0.14	1.1^B.a^ ± 0.03

Significance of the comparisons between treatments (i.e., raw or cooked product with or without application of high-pressure treatment) is indicated by the first superscript (in capital letters), different superscripts within the same row, meaning a significant difference at *p* < 0.05. Significance of comparisons for the same treatment between the two storage times (3 and 22 days) is indicated by the second superscript after the comma (lowercase letters), different superscripts meaning a significant difference at *p* < 0.05. ^#^AMP, adenosine-5′-monophosphate; IMP, inosine-5′-monophosphate; and GMP, guanosine-5′-monophosphate.

Noteworthy, the highest IMP concentration was found in raw cured pork loins treated by HPP at 300 MPa (Table 3), which pinpoints a formation of IMP under these HP conditions. Huijuan et al. (26) found that the activity of adenosine monophosphate deaminase (AMPD), which is responsible for catalyzing the reaction from AMP to IMP, was maximized precisely under 300 MPa, promoting the formation of IMP to 1,250%. Furthermore, HPP at 150 and 300 MPa also improved the sensory quality of meat marinated in soy sauce by promoting the formation of different metabolites, including nucleotides (41). In contrast, treating raw pork loins at a higher pressure, 600 MPa, did not increase IMP compared to the control. Cooked samples did not show any increase in IMP, supposedly, as muscle enzymes responsible for the formation of IMP, namely the enzyme AMPD, must already have been inactive in cooked meat. Moreover, IMP was degraded over storage time in raw pork loins. However, it was not degraded when the raw cured pork loins were treated by HPP at 600 MPa or cooked, likely because these processing conditions resulted in enzyme inactivation of IMP degrading enzymes.

GMP was higher in control raw than in cooked cured pork loins, though the differences were relatively minor. The same explanation for the AMP could be valid here, as GMP was higher in the less processed samples (i.e., no HPP and/or cooking). In addition, GMP was reduced with storage time in raw cured pork loins, whereas it was not degraded in cooked cured pork loins.

Overall, cooking seemed to stop most of the nucleotide breakdown pathway activity, likely via the thermal inactivation of endogenous enzymes. The treatment of meat products by HPP, either raw or cooked, resulted in differences in the nucleotide profiles, and thus, this can affect flavor perception. Based on our results, the umami-taste substance, IMP, might be increased by applying HPP treatments at 300 MPa in raw meat products. The enzyme responsible for catalyzing this reaction is the AMPD, whose activity has been proven to be thermodynamically favored under pressure (26).

### 3.3 Overall discussion

**Figure 3:**
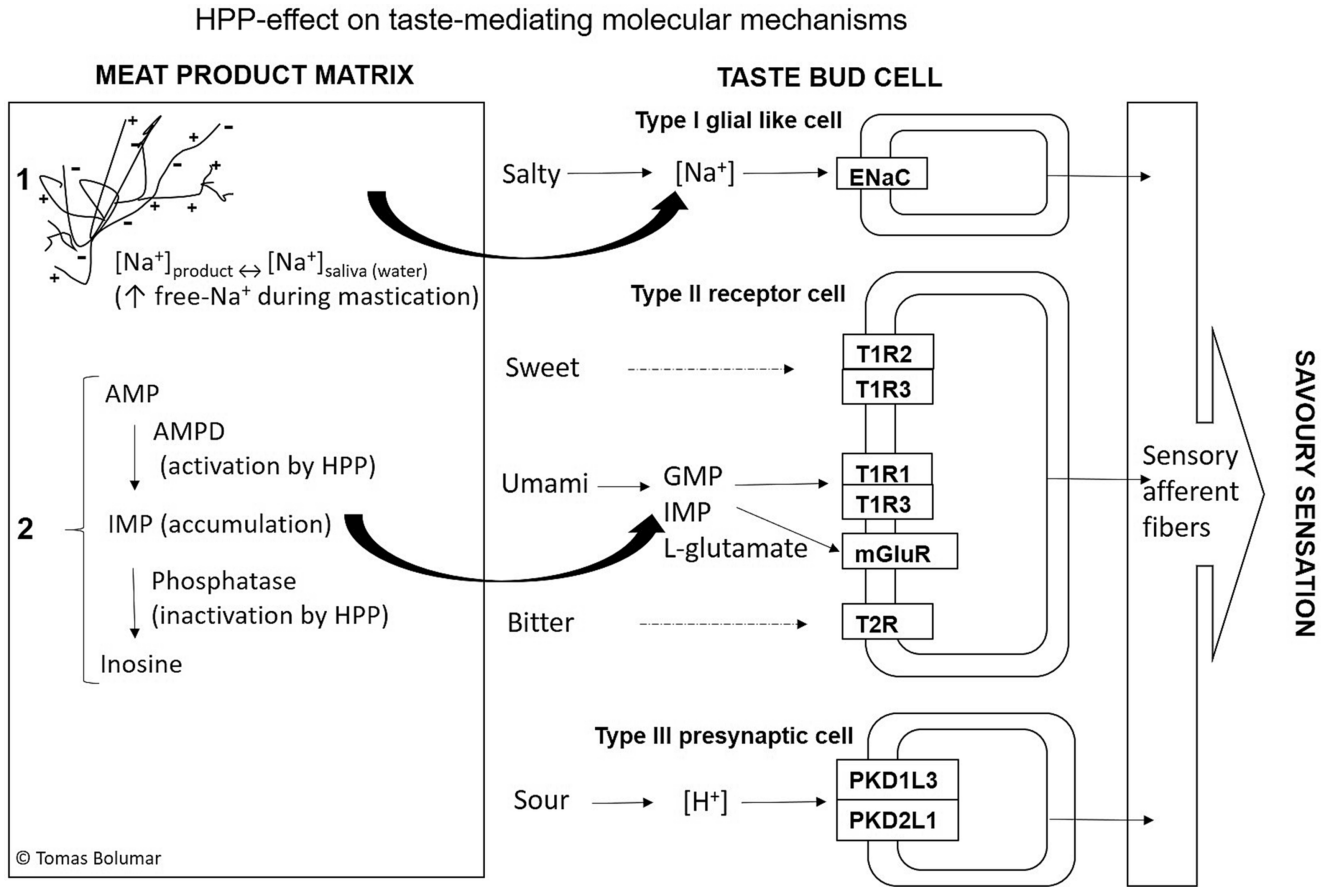
Schematic pathway of the five primary taste sensing transductions in humans [adapted from Kyoto Encyclopedia of Genes and Genomes (KEGG) (43)] and the two hypothetical molecular mechanisms suggested for manipulation of taste intensity through high-pressure processing (HPP) induced changes. Mechanism (1). Modifying electrostatics interactions between meat matrix, primarily proteins, and sodium ions (Na^+^) leads to more free-Na^+^ available to interact with salty-taste receptors. Mechanism (2). Nucleotide breakdown reactions in muscle result in the accumulation of IMP upon pressure treatment [adapted from Huijuan et al. (26)], with subsequent IMP docking on T1R1 and T1R3 umami-taste receptors. HPP, high-pressure processing; AMPD, adenosine monophosphate deaminase; AMP, adenosine-5′-monophosphate (AMP); IMP, inosine-5′-monophosphate; ENaC, epithelial sodium channel; Protein selected ion channels: PKD1L3 and PKDL.

Salt reduction comes with a reduction of flavor intensity that usually leads to reduced consumer acceptability (8). Therefore, the food industry should develop salt reduction strategies that guarantee adequate sensory eating quality. Taste perception is fundamentally formed at the interaction between food components and their docking with taste receptors on the tongue. A proper combination of salty and umami perception in savory products is paramount to defining overall acceptability. Salty taste is mediated via the epithelial sodium channel (ENaC), playing a major role in the perception of salts in saliva (42) (Figure 3). Umami, described as savory, meaty, or brothy, was initially identified by Kikunae Ikeda as the salt of L-glutamic acid in 1908 (44). The umami taste is elicited by the salts of two amino acids, L-glutamic acid (L-Glu) and L-aspartic acid (L-Asp), through binding to umami taste receptors T1R1 + T1R3 (45) and GluR4 (46). Monosodium glutamate (MSG) is widely used to impart umami flavor and enable sodium reductions (47). In addition, AMP, IMP, and GMP are meaty compounds that enhance umami taste by working together with amino acids by intensifying the taste sensation via binding to the same T1R1 + T1R3 receptors (24, 25, 42) (Figure 3).

The interaction of umami and salty perception affects the overall sensation of saltiness. Umami carriers, among others MSG and IMP, effectively enhance saltiness (48). The perception of odor and taste combined results in the overall flavor. Thus, an additional factor in saltiness perception, apart from the presence of specific ions and umami substances and their active concentration, is the interaction between aroma perception and taste sensation in the postnasal cavity. Substances with salty characteristics can significantly enhance sodium chloride perceived intensity and salty taste sensation (49). Therefore, another effective salt reduction strategy is using compounds with salty odor characteristics to enhance salty taste perception. Odor substances imparting salty characteristics enhance the perception of salty flavors. 3-(methylthio)propanal, 1-octen-3-ol, 2,5dimethylpyrazine, and 3-(methylthio) propanol isolated from soy sauce could enhance the salty taste perception (50). An integrative multifactorial saltiness-perception modulation considering (1) ions (salty), (2) umami substances, and (3) volatiles with salty enhancement ability could bring benefits when developing low-sodium foods.

Figure 3 depicts a schematic representation of the suggested hypothetical mechanisms behind the HPP-induced effect on saltiness perception and overall acceptability. First, HPP could induce enough protein denaturation in raw meat products to change electrostatics interactions, leading to more free-Na^+^ for active interaction with the ENaC channel, particularly during mastication. That will intensify the saltiness perception. Second, umami-taste nucleotides like IMP, which can accumulate in HPP-treated products, will interact with T1R1 + T1R3 receptors. In combination with amino acids, this will boost the umami flavor.

The study was a first step to ascertain the HPP effect on the saltiness perception of commercially available meat products, and salt-reduced products beyond the standard levels could be assayed in further trials, where microbiological safety aspects would need to be evaluated. Yet, due to the large variability encountered in the salt content of meat products in food composition tables within a product category, with sausages 1.5–2.7 g/100 g, frankfurter 1.8–2.3 g/100 g and cooked hams 2.3–3.0 g/100 g [USDA Food National Database cited by Inguglia et al. (4)] and own data collected from the German market product monitoring [Max Rubner Institute (51), Table 6 for values of meat products], products with much higher salt content than the employed in this study are currently in the market. Hence, the HPP application already brings a salt reduction potential.

In this sense, however, it is difficult to reasonably predict what will occur under lower/higher salt content. One could forecast that the saltiness perception of products with higher salt content could be less influenced by HPP, as a higher salt concentration could bring about an excess of salt in saliva, giving rise to more salt interacting with taste receptors in the mouth and a possible saturated condition. Thus, the effect on the perception of saltiness could likely be more pronounced and relevant in lower salt-content meat products. In this sense, the salty taste elicited with table salt (NaCl) has a human detection threshold of about 1 ∼ 30 mmol^−1^ (i.e., 0.18 g NaCl/100 g). For reference, many commercially available soups contain NaCl at 100 ∼ 200 mmol^−1^ (i.e., 0.58–1.17 g NaCl/100 g) (52). Of course, the product’s chemical and structural conditions at a micro- and macro-molecular level could influence these generic thresholds and would have to be investigated experimentally on a product-case basis.

The flavor industry utilizes extracts that simultaneously affect saltiness and umami perception, providing solutions tailored to salt-reduced meat products (53). Overall, the content of free-Na^+^ and umami-taste nucleotides in meat products target two different taste receptors, which are fundamental. A better molecular understanding of the thermodynamics and biochemical routes governing the concentration of compounds affecting both receptors can be used to develop meat products with reduced salt content while maintaining similar consumer acceptability and deserve further exploration.

## 4 Conclusion

HPP treatment at 300 and 600 MPa enhanced the perception of saltiness and overall sensory acceptability of raw (uncooked) cured pork loins but not of their cooked counterparts. This modulation of saltiness perception opens a new avenue to boost the taste perception of raw meat products by post-packaging HPP treatments. The causes behind this increase in saltiness perception are currently not well-established. No differences were detected in the *P*_Na+_, which suggests that the kinetics of the release of Na^+^ during mastication could be more related than at the equilibrium time point (as for the *P*_Na+_). HPP at 300 MPa resulted in an increased content of IMP in raw cured pork loins that could also mediate to boost taste intensity. Overall, when reducing the salt (NaCl) content in meat products, HPP could be a technological tool for the industry to ensure food safety and shelf-life and manipulate and enhance product properties of importance, such as saltiness perception and sensory acceptability.

## Data availability statement

The raw data supporting the conclusions of this article will be made available by the authors, without undue reservation.

## Ethics statement

Ethical approval was not required for the studies involving humans because the sensory test was carried out on food products (cured loins) produced, stored and prepared under standard and approved food conditions for human consumption, which comply with current legislation in Germany. The meat to make the food products used in the sensory analysis were sorted and processed in establishments with governmental approval to produce foods for human consumption: the Kulmbach Municipality Slaughterhouse and the Pilot Plant of the Department of Safety and Quality of Meat of the Max Rubner Institute (MRI) in Kulmbach. In Bavaria (Germany), where the MRI’s site in Kulmbach is located, sensory testing of meat and meat products approved for human consumption does not require formal advice from an ethics committee. The studies were conducted in accordance with the local legislation and institutional requirements. The participants provided their written informed consent to participate in this study.

## Author contributions

TB: Conceptualization, Formal analysis, Methodology, Writing – original draft, Writing – review & editing. RL: Formal analysis, Investigation, Writing – review & editing. MP: Formal analysis, Investigation, Writing – review & editing. KT: Formal analysis, Investigation, Writing – review & editing. SM: Conceptualization, Formal analysis, Writing – review & editing. DB: Conceptualization, Funding acquisition, Project administration, Supervision, Writing – review & editing.

## References

[1] World Health Organisation (WHO). (2013). Mapping salt reduction initiatives in the WHO European region. Copenhagen: WHO Regional Office for Europe, 1–51.

[2] Ruusunen M Puolanne E. Reducing sodium intake from meat products. Meat Sci. (2005) 70:531–41. doi: 10.1016/j.meatsci.2004.07.016 22063751

[3] Desmond E. Reducing salt: a challenge for the meat industry. Meat Sci. (2006) 74:188–96. doi: 10.1016/j.meatsci.2006.04.014, PMID: 22062728

[4] Inguglia ES Zhang Z Tiwari BK Kerry JP Burgess CM. Salt reduction strategies in processed meat products – a review. Trends Food Sci Technol. (2017) 59:70–8. doi: 10.1016/j.tifs.2016.10.016

[5] Jaenke R Barzi F Mcmahon E Webster J Jaenke R Barzi F. Consumer acceptance of reformulated food products: a systematic review and meta-analysis of salt-reduced foods. Crit Rev Food Sci Nutr. (2017) 57:3357–72. doi: 10.1080/10408398.2015.1118009, PMID: 26745848

[6] Tamm A Bolumar T Bajovic B Toepfl S Heinz V. Salt (NaCl) reduction in cooked ham by a combined approach of high pressure treatment and the salt replacer KCl. Innovative Food Sci Emerg Technol. (2016) 36:294–302. doi: 10.1016/j.ifset.2016.07.010

[7] Wang J Huang XH Zhang YY Li S Dong X Qin L. Effect of sodium salt on meat products and reduction sodium strategies – a review. Meat Sci. (2023) 205:109296. doi: 10.1016/j.meatsci.2023.109296, PMID: 37562267

[8] Hoppu U Hopia A Pohjanheimo T Rotola-Pukkila M Mäkinen S Pihlanto A. Effect of salt reduction on consumer acceptance and sensory quality of food. Food Secur. (2017) 6:103. doi: 10.3390/foods6120103, PMID: 29186893 PMC5742771

[9] Bajovic B Bolumar T Heinz V. Quality considerations with high pressure processing of fresh and value added meat products. Meat Sci. (2012) 92:280–9. doi: 10.1016/j.meatsci.2012.04.024, PMID: 22608831

[10] Bolumar T Orlien V Sikes A Aganovic K Bak KH Guyon C. High-pressure processing of meat: molecular impacts and industrial applications. Compr Rev Food Sci Food Saf. (2021) 20:332–68. doi: 10.1111/1541-4337.12670, PMID: 33443800

[11] Nuygen M Arvaj L Balamurugan S. The use of high pressure processing to compensate for the effects of salt reduction in ready-to-eat meat products. Crit Rev Food Sci Nutr. (2022) 1-15:1–15. doi: 10.1080/10408398.2022.2124398, PMID: 36106480

[12] Clariana M Guerrero L Sarraga C Diaz I Valero A Garcia-Regueiro JA. Influence of high pressure application on the nutritional, sensory and microbiological characteristics of sliced vacuum packed dry-cured ham. Effects along the storage period. Innovative Food Sci Emerg Technol. (2011) 12:456–65. doi: 10.1016/j.ifset.2010.12.008

[13] Fuentes V Estevez M Grebol N Ventanas J Ventanas S. Application of time-intensity method to assess the sensory properties of Iberian dry-cured ham: effect of fat content and high-pressure treatment. Eur Food Res Technol. (2014) 238:397–408. doi: 10.1007/s00217-013-2113-8

[14] Fulladosa E Serra X Gou P Arnau J. Effects of potassium lactate and high pressure on transglutaminase restructured dry-cured hams with reduced salt content. Meat Sci. (2009) 82:213–8. doi: 10.1016/j.meatsci.2009.01.013, PMID: 20416756

[15] Saccani G. Parolari G. Tanzi E. Rabbuti S. (2004). Sensory and microbiological properties of dried hams treated with high hydrostatic pressure. Proceedings of the 50th ICoMST, Helsinki, Finland.

[16] Pietrasik Z Gaudette NJ Johnston SP. The impact of high hydrostatic pressure on the functionality and consumer acceptability of reduced sodium naturally cured wieners. Meat Sci. (2017) 129:127–34. doi: 10.1016/j.meatsci.2017.02.020, PMID: 28284123

[17] Zhou Y Watkins P Oiseth S Cochet-Broch M Sikes AL Chen C. High pressure processing improves the sensory quality of sodium-reduced chicken sausage formulated with three anion types of potassium salt. Food Control. (2021) 126:108008. doi: 10.1016/j.foodcont.2021.108008

[18] Zhu Y Yan Y Yu Z Wu T Bennett LE. Effects of high pressure processing on microbial, textural and sensory properties of low-salt emulsified beef sausage. Food Control. (2022) 133:108596. doi: 10.1016/j.foodcont.2021.108596

[19] Phan VA Yven C Lawrence G Chabanet C Reparet JM Salles C. In vivo sodium release related to salty perception during eating model cheeses of different textures. Int Dairy J. (2008) 18:956–63. doi: 10.1016/j.idairyj.2008.03.015

[20] Puolanne E Halonen M. Theoretical aspects of water-holding in meat. Meat Sci. (2010) 86:151–65. doi: 10.1016/j.meatsci.2010.04.038, PMID: 20627421

[21] Aristoy M.C. Toldra F. (2009). Nucleotides and its derived compounds. Leo M. Nollet L. Toldra Fidel, Handbook of muscle foods analysis. 279–298). Boca Raton, FL: CRC Press, Taylor and Francis.

[22] Elmore JS Mottram DS. Flavour development in meat In: Kerry JP Ledward AD, editors. Improving the sensory and nutritional quality of fresh meat. Cambridge, England: Woodhead Publishing Limited (2009). 111–46.

[23] Maga JA. Umami flavor of meat In: Shahidi F, editor. Flavour of meat and meat products. New York: Chapman and Hall (1994). 98–115.

[24] Mouritsen OG Khandelia H. Molecular mechanism of the allosteric enhancement of the umami taste sensation: dynamics of the umami receptor. FEBS J. (2012) 279:3112–20. doi: 10.1111/j.1742-4658.2012.08690.x, PMID: 22764741

[25] Zhang Y Venkitasamy C Pan Z Wang W. Recent developments on umami ingredients of edible mushrooms – a review. Trends Food Sci Technol. (2013) 33:78–92. doi: 10.1016/j.tifs.2013.08.002

[26] Huijuan Z Jian P Juan L Xiaoxiao X. High-pressure effects on the mechanism of accumulated inosine 5′-monophosphate. Innovative Food Sci Emerg Technol. (2018) 45:330–4. doi: 10.1016/j.ifset.2017.12.005

[27] Utama DT Lee SG Baek KH Jang A Pak JI Lee SK. Effects of high-pressure processing on taste-related ATP breakdown compounds and aroma volatiles in grass-fed beef during vacuum aging. Asian-Australasian J Anim Sci. (2018) 31:1336–44. doi: 10.5713/ajas.17.0677, PMID: 29531191 PMC6043434

[28] Boisard L Andriot I Martin C Septier C Boissard V Salles C. The salt and lipid composition of model cheeses modifies in-mouth flavour release and perception related to the free sodium ion content. Food Chem. (2013) 145:437–44. doi: 10.1016/j.foodchem.2013.08.049, PMID: 24128499

[29] Lauverjat C De Loubens C Déléris I Tréléa IC Souchon I. Rapid determination of partition and diffusion properties for salt and aroma compounds in complex food matrices. J Food Eng. (2009) 93:407–15. doi: 10.1016/j.jfoodeng.2009.02.003

[30] Saint-eve A Lauverjat C Magnan C Déléris I Souchon I. Reducing salt and fat content: impact of composition, texture and cognitive interactions on the perception of flavoured model cheeses. Food Chem. (2009) 116:167–75. doi: 10.1016/j.foodchem.2009.02.027

[31] Wirth F. Reducing the fat and sodium content of meat-products – what possibilities are there. Fleischwirtschaft. (1991) 71:294–7.

[32] Flores M. The eating quality of meat: flavor In: Toldra F, editor. Lawrie's meat science. UK: Woodhead Publishing is an imprint of Elsevier (2017). 383–417.

[33] Aaslyng MD Meinert L. Meat flavour in pork and beef–From animal to meal. Meat science (2017). 132, 112–117. 28457663 10.1016/j.meatsci.2017.04.012

[34] Vaskoska R Ha M Ong L Chen G White J Gras S. Myosin sensitivity to thermal denaturation explains differences in water loss and shrinkage during cooking in muscles of distinct fibre types. Meat Sci. (2021) 179:108521. doi: 10.1016/j.meatsci.2021.108521, PMID: 33964804

[35] Zheng H-B Xu B-C Xu X-L Li C Bolumar T Zhen Z-Y. Gelation of chicken batters during heating under high pressure. Innov Food Sci Emerg Technol. (2021) 74:102848. doi: 10.1016/j.ifset.2021.102848

[36] Bak KH Bolumar T Karlsson AH Lindahl G Orlien V. Effect of high pressure treatment on the color of fresh and processed meats: a review. Crit Rev Food Sci Nutr. (2019) 59:228–52. doi: 10.1080/10408398.2017.1363712, PMID: 28846443

[37] Bak KH Lindahl G Karlsson AH Orlien V. Effect of high pressure, temperature, and storage on the color of porcine *longissimus dorsi*. Meat Sci. (2012) 92:374–81. doi: 10.1016/j.meatsci.2012.02.002, PMID: 22749540

[38] Ruusunen M Simolin M Puolanne E. The effect of fat content and flavor enhancers on the perceived saltiness of cooked 'bologna-type' sausages. J Muscle Foods. (2001) 12:107–20. doi: 10.1111/j.1745-4573.2001.tb00303.x

[39] Tobin BD O'Sullivan MG Hamill RM Kerry JP. The impact of salt and fat level variation on the physiochemical properties and sensory quality of pork breakfast sausages. Meat Sci. (2013) 93:145–52. doi: 10.1016/j.meatsci.2012.08.008, PMID: 23022579

[40] Yamaguchi S Yoshikawa T Ikeda S Ninomiya T. Measurement of the relative taste intensity of some L-α-amino acids and 5′-nucleotides. J Food Sci. (1971) 36:846–9. doi: 10.1111/j.1365-2621.1971.tb15541.x

[41] Yang Y Ye YF Wang Y Sun Y Pan D Cao JX. Effect of high pressure treatment on metabolite profile of marinated meat in soy sauce. Food Chem. (2018) 240:662–9. doi: 10.1016/j.foodchem.2017.08.006, PMID: 28946326

[42] Spaggiari G Di Pizio A Cozzini P. Sweet, umami and bitter taste receptors: state of the art of in silico molecular modeling approaches. Trends Food Sci Technol. (2020) 96:21–9. doi: 10.1016/j.tifs.2019.12.002

[43] Kyoto Encyclopaedia of Genes and Genomes (KEGG). (2019). Taste transduction – *Homo sapiens* (human). Available at: https://www.genome.jp/kegg-bin/show_pathway?map=hsa04742&show_description=show (Accessed April 3, 2020)

[44] Ikeda K. New seasonings. Chem Senses. (2002) 27:847–9. doi: 10.1093/chemse/27.9.847 12438213

[45] Nelson G Chandrashekar J Hoon MA Feng L Zhao G Ryba NJP. An amino-acid taste receptor. Nature. (2002) 416:199–202. doi: 10.1038/nature726 11894099

[46] Chaudhari N Landin AM Roper SD. A metabotropic glutamate receptor variant functions as a taste receptor. Nat Neurosci. (2000) 3:113–9. doi: 10.1038/72053, PMID: 10649565

[47] Maluly HDB Arisseto-Bragotto AP Reyes FGR. Monosodium glutamate as a tool to reduce sodium in foodstuffs: technological and safety aspects. Food Sci Nutr. (2017) 5:1039–48. doi: 10.1002/fsn3.499, PMID: 29188030 PMC5694874

[48] Sun X Zhong K Zhang D Shi B Wang H Shi J. The enhancement of the perception of saltiness by umami sensation elicited by flavor enhancers in salt solutions. Food Res Int. (2022) 157:111287. doi: 10.1016/j.foodres.2022.111287, PMID: 35761595

[49] Vinitha K Sethupathy P Moses JA Anandharamakrishnan C. Conventional and emerging approaches for reducing dietary intake of salt. Food Res Int. (2022) 152:110933. doi: 10.1016/j.foodres.2021.110933, PMID: 35181101

[50] Zhou T Feng Y Thomas D. Enhancement of saltiness perception by odorants selected from Chinese soy sauce: a gas chromatography/olfactometry associated taste study. Food Chem. (2020) 335:127664. doi: 10.1016/j.foodchem.2020.127664 32739820

[51] Max Rubner Institute. (2020). Produktmonitoring 2020. Ergebnisbericht. Available at: https://www.mri.bund.de/fileadmin/MRI/Institute/EV/MRI-Produktmonitoring-2020_Ergebnisbericht-final.pdf (Accessed January 31, 2024)

[52] Roper SD. Taste: mammalian taste bud physiology. Reference Module Neurosci Biobehav Psychol. (2017):887–93. doi: 10.1016/B978-0-12-809324-5.02908-4

[53] Wang S Tonnis BD Wang ML Zhang S Adhikari K. Investigation of monosodium glutamate alternatives for content of umami substances and their enhancement effects in chicken soup compared to monosodium glutamate. J Food Sci. (2019) 84:3275–83. doi: 10.1111/1750-3841.14834, PMID: 31602667

